# Activation and Evasion of Innate Antiviral Immunity by Herpes Simplex Virus

**DOI:** 10.3390/v1030737

**Published:** 2009-11-05

**Authors:** Jesper Melchjorsen, Sampsa Matikainen, Søren R. Paludan

**Affiliations:** 1 Department of Infectious Diseases, Aarhus University Hospital, Skejby, Denmark; 2 Unit of Excellence for Immunotoxicology, Finnish Institute of Occupational Health, Topeliuksenkatu 41 A, 00250 Helsinki, Finland; E-Mail: sampsa.matikainen@ttl.fi; 3 Department of Medical Microbiology and Immunology, Aarhus University, The Bartholin Building, 8000 Aarhus C, Denmark; E-Mail: srp@microbiology.au.dk

**Keywords:** herpes simplex virus, vasion, innate, interferon, viral immunity

## Abstract

Herpes simplex virus (HSV), a human pathogenic virus, has evolved several strategies to evade the production and function of interferons (IFNs) and cytokines generated by the innate immune system to restrict the virus. Equilibrium exists between the virus and the immune response, and a shift in this delicate balance either restricts the virus or enhances virus spread and tissue damage. Therefore, understanding of the cytokine response generated after HSV infection and the underlying virus-cell interactions is essential to improve our understanding of viral pathogenesis. This review summarizes the current knowledge on induction and evasion of the innate immune response by HSV.

## 1. Introduction

Herpes simplex virus (HSV) is a very common human pathogenic virus. Clinically, HSV infection may give rise to gingivostomatitis, cold sore, keratitis, encephalitis, and genital herpes [1]. The diseases are normally self-limiting but in immunocompromised individuals, such as newborns, transplantation patients, and AIDS-patients, the virus may cause devastating dissimilated infections and encephalitis [1].

Type I interferons (IFN)s, also known as IFN-α/β, and to a lesser extent type III IFNs (IFN-λ1–3) are important for antiviral response against HSV [2–5]. In addition to their direct antiviral effect, IFNs together with proinflammatory cytokines are important signalling molecules for activation and attraction of leukocytes to the site of infection.

Pathogens are a highly diverse group of microbes and viruses. Their detection is therefore a major challenge to the immune system. Host defense against infections is initiated by the innate immune system, operating on the basis of general pathogen features via pattern recognition receptors (PRRs). PRRs detect pathogen-associated molecular patterns (PAMPs) and signal the presence of infection to the host, activating host defense, including antiviral (IFN-α/β) and pro-inflammatory cytokine (interleukin-1β (IL-1β), IL-6, IL-18 and tumor necrosis factor α (TNF-α) production. In addition, PRRs often initiate programmed cell death (apoptosis) of infected cells.

Toll-like receptors (TLRs) are the best characterized family of PRRs that recognize PAMPs in mammals. There are ten characterized TLRs in humans and they are a class of membrane receptors that sense microbes either in the extracellular space or in intracellular endolysosomal compartments. The second important group of PRRs that have an important role in recognition of HSV infection are retinoid acid inducible gene-I (RIG-I)-like receptors (RLRs), including RIG-I and melanoma differentiation-associated gene-5 (MDA-5). They are focused on detecting viral genomic RNA or its replication intermediates in the cytoplasm [6]. Finally a third pathway is presumed to recognize DNA in the cytoplasm, potentially also genomic DNA from HSV. One suggested DNA receptor is DNA-dependent activator of IFN Regulatory Factors (DAI) that is able to activate type I IFN production in response to cytosolic DNA [7]. However, the role of DAI in the *in vivo* recognition of cytoplasmic DNA has remained controversial. Recently, cytosolic DNA-dependent RNA polymerase III has been shown to recognize cytosolic DNA and linked to recognition of HSV and the production of IFN-β [8,9]. Further studies are required to reveal the specific role of DAI, RNA polymerase III and other putative other DNA receptors in activation of innate immune response during virus infection in humans.

**Figure 1.**
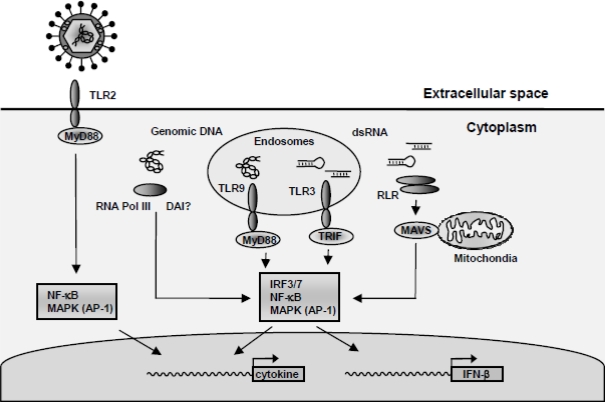
Activation of antiviral and proinflammatory responses during HSV infections. HSV is recognized by cells through several mechanisms. (i) Virus-receptor interactions can induce intracellular signal transduction leading to cytokine expression. Potential recognition receptors include TLR2 and the virus entry mediators HVEM. (ii) Viral genomic DNA is recognized by TLR9 and a DNA receptor in the cytoplasm, including RNA polymerase III. (iii) Accumulating of viral dsRNA during viral replication is potentially sensed through several mechanisms, including TLR3 and the RLRs. (iv) Recognition of the virus results in activated signalling pathways, such as the MAPK pathway (AP-1), NF-κB and IRF3/7 regulating the expression of IFN-β and other cytokines.

To mount an efficient antiviral immune response the cell must recognize the virus and activate a number of signalling pathways, including Nuclear Factor kappa B (NF-κB), IFN Regulatory Factors (IRFs) and Mitogen-Activated Protein Kinase (MAPK) pathways. As summarized in Figure 1, the recognition of HSV includes (i) recognition of viral surface glycoprotein via either TLR2 or and yet unidentified receptor [10–12], (ii) recognition of HSV viral DNA by TLR9 in endosomes, or in the cytosol via either RNA polymerase III or potentially DAI [7,9,13–16], and (iii) the recognition of virally-derived double-stranded (ds)RNA recognized by RLRs [16]. Finally, TLR3 plays a role in restricting HSV infection evidenced by a recent study showing that humans that bear mutations in TLR3 are predisposed to HSV-associated encephalitis [17].

To gain time, and potentially also to allow chronic infection, HSV has evolved numerous strategies to evade IFN signalling at different stages, including directly affecting JAK/STAT signalling and interactions with the IFN-induced antiviral proteins Protein Kinase R (PKR) and the 2′–5′-Oligoadenylate Synthethase (2′–5′-OAS)/RNaseL system. Furthermore, HSV has means of escaping cytokine and IFN production via inhibition of the transcription factors NF-κB and IRF3 and IRF7 signalling pathways, reduction of cytokine mRNA stability and interfering with translation.

This review presents the current knowledge on antiviral escape mechanisms employed by HSV.

## 2. Herpes Simplex Virus and Replication of the Virus Genome

HSV-1 and HSV-2 are closely related, enveloped, nuclear replicating, double-stranded (ds)DNA viruses belonging to the subgroup of *alphaherpesvirinae.* The two subtypes share 83% homology in the protein coding regions of the genome [18], and exhibit many similar biological functions [1]. Because of similarities between the two subtypes, no distinction between HSV-1 and HSV-2 will be made in the following sections. The reader is encouraged to consult the primary literature for data on the individual HSV subtypes.

**Figure 2.**
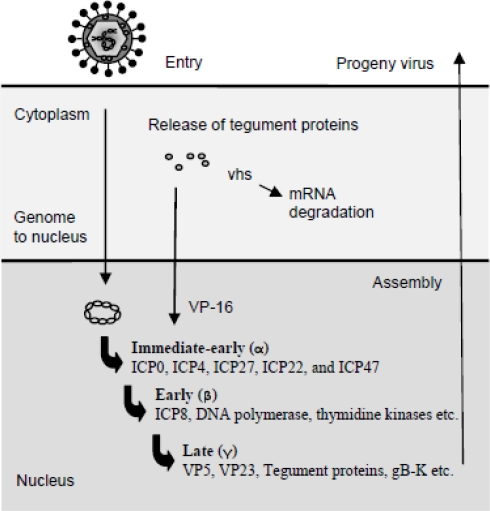
The replication cycle of HSV. After virus entry, the viral capsid is transported to the nucleus and the genome released into the nucleus through nuclear pores. Viral tegument proteins released into the cell support IE gene transcription or mediate takeover of the cell. The IE proteins are mainly trans-activators that enhance the expression of E genes, which primarily encode enzymes involved in virus DNA replication. Eventually, the L genes are expressed and new virus particles are assembled, matured, and released from the cell, either by cell lysis, endocytosis, or cell-cell fusion.

The transcription of viral genes is tightly regulated and has two key features. First, herpes virus proteins fall into groups, whose synthesis is coordinately regulated, *i.e.*, all genes within a given group are expressed sequentially starting with the immediate early (IE) gene group, followed by the early (E) gene group, and finally the late (L) group (Figure 2). The sequential expression occurs because IE proteins are required for expression of the E proteins, and E proteins generally are essential for viral genome replication, which is a prerequisite for the expression of L proteins [1]. The tegument protein VP16 associates with cellular transcription factors to promote the transcription of the five IE mRNAs encoding infected cell protein 0 (ICP0), ICP4, ICP22, ICP27, and ICP47. The IE genes are expressed in the presence of inhibitors of protein synthesis. The four IE proteins, ICP0, 4, 22, and ICP27, are transcriptional and translational regulators of the E and L genes, as well as host genes, whereas ICP47 interferes with the transporter associated with antigen presentation (TAP)1/TAP2 complex and hence antigen presentation through major histocompatibility complex class I (MHC I) [19]. Later sections will further address the evasion strategies employed by HSV. Besides being a positive regulator of IE and L genes, ICP4 also inhibits expression of the IE genes. ICP4’s negative regulation of IE genes provides a halt of IE gene expression during later stages of viral infection. Accumulation of ICP4 and ICP27 initiates the expression of E genes, which primarily encode proteins involved in DNA replication. E gene products include scavenger enzymes, such as the viral thymidine kinase and ribonucleotide reductase, as well as enzymes directly involved in DNA replication, such as the viral DNA polymerase and DNA helicase. Finally, the L genes are made after DNA replication has occurred. These include structural proteins required for the progeny viral particles, as well as VP16 and vhs, both of which are incorporated into the viral tegument. The capsid is assembled in the nucleus and takes up individual DNA genomes once the capsid is fully assembled [20]. Subsequently, the capsids associate with the tegument proteins and bud through the viral glycoprotein-containing nuclear membrane. The virions are hereafter incorporated into vesicles and released by exocytosis. Alternatively, the virus is released by cell lysis or spread by syncytia formation [1].

## 3. Antiviral IFN Response during Early HSV Infection

The first response in a HSV-infected cell is an inflammatory reaction that includes secretion of antiviral substances, such as defensins and nitric oxid and most importantly the production of cytokines, including IFNs and chemokines. The aim of the initial innate immune response is to limit spread of the infection and, if possible, to eliminate the pathogen. The secreted substances activate and attract immune cells and thus help to organize an effective antiviral response.

The type I IFNs IFN-α/β are key cytokines produced within the very first hours after HSV-infection [15,21]. IFN-α/β are important in direct control and inhibition of HSV replication [2,22,23], and this occurs in synergy with IFN-γ [24]. *In vivo* studies show that IFN-α/β alone can control early HSV infection independently of natural killer (NK) cell and lymphocyte activity [25]. Experiments in mice also demonstrate the importance of the IFN system in the antiviral defense against HSV infection because uncontrolled virus replication is seen in mice genetically lacking functional IFN-α/β-receptors or when mice are administered neutralizing antibodies against IFN-α/β [3,26]. The studies in mice have been supported by the recording that patients lacking functional STAT1, an essential components of the IFN pathways, are more prone to HSV encephalitis [4]. Furthermore, IFN-α/β is part of a positive feedback loop that amplifies the cytokine response during HSV infection [27]. Though studies have established an important role for IFN-α/β in antiviral defense, it is difficult to determine whether IFN-α/β’s contribution to viral clearance is primarily mediated by direct antiviral mechanisms or by a indirect immunoregulatory mechanisms.

IFN exerts its activities through induction of hundreds of IFN-stimulated genes (ISGs) [28]. ISGs especially important in antiviral defense are the PKR and the 2′–5′–OAS/RNAse L system (alternatively referred to as the 2′–5′A system). PKR is an important mediator of resistance against HSV because the absence of PKR in murine cells enhances HSV replication [29]. In addition, the HSV-induced expression of IFN-α/β and the proinflammatory chemokine RANTES is dependent on functional PKR [15,30]. The 2′–5′A system seems to play an important role after eye infections, since mice lacking RNAseL and thus the 2′–5′A system have higher mortality rates and display more severe disease progression [31]. In addition, studies in epithelial cells have revealed that IFN-α and especially IFN-β suppress HSV-1 replication through an RNAseL-dependent pathway [32]. It has also been shown that 2′–5′-OAS is a potent inhibitor of HSV replication in BHK cells [33]. As will be discussed later, the activity of PKR and the 2′–5′ A system is counteracted by HSV emphasizing the importance of PKR and the 2′–5′ A system in the innate response against HSV. Additional important ISGs produced during virus infections are chemokines, including IFN-γ-inducible protein of 10 kDA (IP-10, CXCL10), the transcription factor IRF7, and several cellular pattern recognition receptors (PRRs), such as the TLRs and the RLRs [34–39].

Type III IFNs IL-29 (IFN-λ1) and IL-28 (IFN-λ2/3) have been described to possess antiviral effects against HSV [27,40]. IFN-λs activate the same JAK/STAT signalling pathways as type I IFNs, induce resistance to viral infection in several human cell lines, and upregulate expression of ISGs, such as MHC class I antigen, MxA, and 2′–5′OAS [41,42]. IFN-λs are expressed after infection with several RNA viruses and treatment with dsRNA [37,41–43]. Recent data have shown that HSV induces expression of IFN-λs in both human monocyte-derived macrophages and DCs and in murine pDCs and cDCs [5,27]. In addition, both IFN-λ and IFN-α/β are part of a positive feedback loop that greatly enhances the expression of proinflammatory cytokines, chemokines, and IFNs after HSV infection [27]. Type III IFN inhibits HSV IE gene expression with comparable effect to IFN-α/β implying that IFN-λ has direct antiviral effect against HSV [27,40]. Recent studies examining the role of IFN-λ in genital and generalized HSV-2 infection in mice, revealed that IFN-λR knockout mice did not differ from wild-type mice in their ability to clear HSV infection [5]. Nevertheless, it would be interesting to gain insight into the role of IFN-λs during HSV infection in humans, and to establish whether the type III IFNs will have a role in the treatment of HSV infections.

In addition to type I and type III IFN, IFN-γ is a prominent mediator of antiviral immunity against HSV infection [24,44,45]. Studies show that IFN-γ-mediated control of HSV in murine macrophages correlates with production of nitric oxide [44] and that IFN-γ together with IFN-α/β and IL-12 is very important in control of HSV infection [24,25,46]. Furthermore, IFN-γ reduces reactivation of HSV from the neurons [45]. IFN-γ is mainly produced by T and NK cells [47], but also other cells, including macrophages, γδT cells, and DCs, produce IFN-γ after stimulation with IL-12 and IL-18 or after HSV infection [48–52]. Besides its direct antiviral activity, IFN-γ also plays a key role in initiating and amplifying the immune response. IFN-γ for instance, synergizes with HSV to induce the expression of proinflammatory cytokines, such as TNF-α, IL-6, and IL-12 p40 [53,54]. Moreover, IFN-γ indirectly enhances cellular recognition of the virus and subsequent cytokine production through induced expression of potential virus PRRs, such as TLRs and RLRs [36,39,55].

## 4. HSV-Activated Signaling Pathways

The outcome of the infection is often determined by the signal transduction pathways activated. The first line of defense against virus infections requires activation of multiple signal transduction pathways, including NF-κB, MAPK pathways, and IRFs [56]. However, viruses are extremely well adapted to their host and part of this adaption is caused by virus manipulation of host signaling. HSV modulates the signaling pathways to facilitate viral gene expression and enhance viral replication, but may also down-regulate specific pathways to evade immune responses and improve survival in the host cell. During early HSV infection, NF-κB, MAPK and IRF pathways are activated [27,57]. The activated signaling pathways may have dual purposes; both activating cytokine expression and viral replication [58]. It is worth noting that the mechanism through which HSV is recognized and stimulates cellular gene expression is cell type dependent [16]. As will be discussed later the cell-dependency also seems to apply for HSV evasion strategies.

HSV does not directly activate JAK/STAT signalling pathways. JAK/STAT pathways are, nevertheless, very important for development of the proinflammatory response after HSV infection. Many HSV-induced cytokines, including IFN-α/β, IFN-γ, IL-6, and IL-12, mediate phosphorylation of JAK proteins and subsequent phosphorylation and activation of STAT proteins [59]. The activated STAT proteins dimerize, translocate to the nucleus, and activate transcription through binding to the gamma activation site or after association with IRF9 (p48) binding to IFN-stimulated response elements [59]. STAT1 is a central player for IFN-α/β and IFN-γ-activated signal transduction and activity evidenced by the fact that mice and humans deficient in STAT1 are very susceptible to HSV infections, as discussed above [60].

## 5. Evasion of the Innate Immune System by HSV

**Figure 3.**
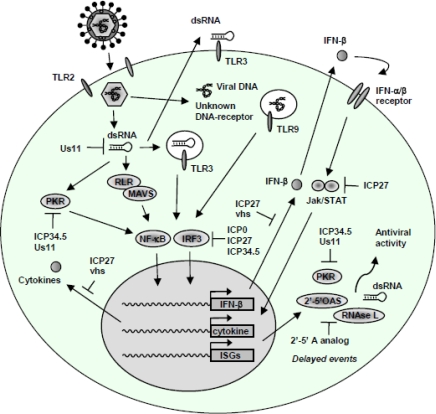
Innate immune evasion mechanisms of HSV. Several countermeasures against the host IFN and proinflammatory response have been established by HSV. For instance, the IRF3 pathway is inhibited by ICP0, ICP27, and ICP34.5, through distinct mechanisms. Also, the HSV L gene products ICP34.5 and Us11 inhibit PKR activation, with Us11 exerting its action by direct binding to dsRNA, for which reason Us11 potentially also blocks activation of TLR3 and RLRs. The 2′–5′-OAS/RNAse L system is inhibited by 2′–5′ A analogs. JAK/STAT signalling and the production of IFN-α/β is counteracted by a mechanism involving ICP27.

Like most viruses, HSV has numerous countermeasures to overcome the host innate defences including several anti-IFN and anti-cytokine weapons and the inhibition of apoptosis. The discussion below will focus on the current knowledge on interaction between HSV and the host response. Figure 3 and Table 1 summarize the strategies employed by HSV to evade the innate immune system.

### 5.1. Evasion of IFN signalling and IFN effector functions

HSV has evolved numerous mechanisms to subvert and repress the IFN-α/β response. Besides the antiviral properties of IFN-α/β, type I IFN also induces expression of antiviral proteins, such as PKR and the 2′–5′ A system. HSV counteracts the production of IFN, diminishes IFN-signalling, and blocks the actions of PKR and activation of the 2′–5′ A system through several viral products, including ICP0, ICP27, ICP34.5 and vhs.

PKR plays an important role in resistance against HSV infection [29]. Moreover, HSV-induced expression of the chemokine RANTES and type I IFNs proceed through a PKR-dependent mechanism [15,30]. Therefore it is not surprising that HSV has evolved at least two mechanisms to counteract the activities of the kinase. The HSV L protein ICP34.5 counteracts PKR activity by recruiting the cellular protein phosphatase 1α, which reverses the PKR-mediated phosphorylation of eIF2α [61,62]. In addition, the viral RNA-binding protein Us11 inhibits PKR through sequestering of dsRNA [62,63] and by direct binding to PKR [64]. Both the Us11 and ICP34.5 gene products are needed to functionally overcome the effect of IFNs [65] and ICP34.5 plays an important role for infection of neurons [66]. Furthermore, Us11 is important for viral inhibition of the 2′–5′-A system [67]. Knowing that Us11 binds to dsRNA, it will be highly interesting to define the role of Us11 in the context of dsRNA recognition via TLR3 or RLR.

In addition, it has been shown HSV inhibits 2′–5′ A/RNAse L-independent rRNA degradation by a mechanism involving ICP0 [68] and one study has shown that HSV uncouples this IFN-inducible mechanism through produced 2′–5′ A analogues [69].

The multifunctional viral IE proteins ICP0 and ICP27 both modulate the immune response generated against HSV infections in numerous ways. Most importantly, ICP0 renders HSV relatively resistant to IFN-α/β and to inhibits the activation of ISGs, and this is in part mediated by inhibiting nuclear translocation of IRF3 [70–74]. ICP0 has been shown to be important for the virus to overcome STAT1-mediated antiviral activity [75]. This finding is, however, in contrast to recent publications showing that STAT1 and IRF3 does not play essential roles in the repression of HSV lacking ICP0 [76,77]. The differences may however, be a matter of differences in experimental setup, including multiplicity of infection used and the assays performed. Recently, it was shown that ICP27 is necessary and sufficient for inhibition of IFN-induced STAT1 phosphorylation and partly for inhibition of STAT1 translocation to the nucleus [78].

ICP27 also inhibits the production of IFN-α/β evidenced by the fact that virus lacking functional ICP27 induces higher levels of IFN-α/β in HeLa cell and higher levels of IFN-α/β and IFN-λs in human macrophages [27,113]. ICP27 seems to inhibit induction IFN and cytokines in macrophages via inhibition of NF-κB and IRF3 activation [27]. This is in accordance with results from 293T cells showing that ICP27 inhibits NF-κB by stabilization of its inhibitor component Inhibitory kappa B alpha (IκBα [114]. Nonetheless, the inhibitory function of ICP27 might be cell specific, since reports have shown that ICP27 alone is sufficient to activate NF-κB and MAPK pathways in Hela cells and CV-1 fibroblast-like cells [115–117].

Vhs is a multifunctional protein and an important determinant for HSV virulence [118]. The protein participates in many different immunmodulatory mechanisms, including regulation of IFN-α/β production both in mice and murine fibroblasts after HSV infection [80,81]. In addition, HSV deficient in vhs is highly susceptible to IFN-α/β [84]. This may be explained by recent findings showing that vhs interferes with IFN-signalling. Specifically, vhs was shown to inhibit JAK/STAT signalling and IRF7 expression, evidenced by findings showing that a vhs-deficient virus only weakly inhibited IFN-signalling and expression of IRF7 [82]. The HSV- and vhs-mediated suppression of JAK/STAT signalling resulted in impaired expression of IRF7 and IFN-α [82]. IRF7 is essential for development of an effective antiviral *in vivo* response against HSV [119]. Recent data suggest the ICP27 is major player in HSV-mediated inhibition of IFN signalling, since extopic expression of ICP27 efficiently blocked STAT1 phosphorylation and subsequent translocation to the nucleus [78]. Finally, HSV indirectly inhibits IFN signalling via upregulation of suppressor of cytokine signalling (SOCS) 1 and 3 in keratinocytes and a human amnion cell line, respectively [82,120]. Of eight characterized SOCS proteins (SOCS1-7 and cytokine-induced SH2 protein (CIS)), only SOCS1 and 3 has been shown to inhibit IFN-signalling. The assays and conclusions are, however, complicated by the fact that SOCS expression is part of the general negative feedback loop of IFN signalling. Whether HSV-induced SOCS1 and 3 expression is a general mechanism of HSV resistance to IFN signalling remains to be determined.

### 5.2. Inhibition of cytokine and IFN gene transcription and translation

Many cellular genes are affected by HSV infection. Initially, HSV infection may induce an antiviral response independent of viral replication. Next, this early proinflammatory response may be disarmed dependent on viral gene products [27,91,121–123]. In the following section, the immunomodulatory mechanisms of HSV are discussed. The mechanisms of counteracting and regulating cell gene expression are numerous and include HSV-mediated transcriptional repression, impaired mRNA stability, and inhibition of translation.

Viral ICP27 is a well-described candidate for HSV-mediated immune evasion because it reduces the abundance of host mRNAs through inhibition of pre-mRNA splicing [88,124] and reduction of RNA stability [91]. Furthermore, ICP27 represses host cytokine gene expression [27], and together with vhs mediates efficient inhibition of host protein synthesis [125,126]. In addition to ICP27, also ICP4 has been linked to a decrease in cytokine mRNA stability [91]. HSV-mediated repression of host gene transcription includes an ICP27-dependent impairment of RNA polymerase (RNAP)II transcription, which negatively regulates some cellular genes [122]. One might speculate that a direct interaction between ICP27 and RNAPII [127] regulates the transcriptional function of the enzyme. Whether HSV-inhibition of RNAPII transcription plays a direct role in regulating cytokine gene expression still needs to be defined.

IRF3 is an essential player in regulating transcription of IFN-β and many proinflammatory cytokines and chemokines. The importance of IRF3 in anti-HSV defense is underscored by the fact that HSV interferes with IRF3 activation in multiple ways [27,72–74]. ICP0 has been shown to interfere with IRF3 and IRF7 activation of ISGs [72,74]. Interference with IRF3 includes ICP0- and ICP27-mediated inhibition of IRF3 nuclear accumulation, which at least for ICP0 is mediated through direct binding to IRF3 [27,71–73]. Finally, ICP0 has recently been shown to modulate TLR-mediated immune responses blocking activation of NF-κB and the MAPK c-Jun NH2-terminal kinase (JNK) [87]. In addition to its transcriptional regulatory role, ICP0 may potentially also affect the translational machinery through its interaction with elongation factor-1δ [128].

Besides the role of vhs in evasion of IFN activity and degradation of mRNA, vhs also reduces the production of IL-1β, IL-8 and CCL3 in human monocytes and macrophages after HSV infection [84]. Among the cytokines investigated especially IL-8 is negatively regulated, potentially explaining why this chemokine is rarely expressed after HSV infections. The vhs-mediated inhibition of CXCL8 production is of great interest, taking into account that IL-8 is reported an effective adjuvant for DNA vaccines against HSV in mice [129]. Correspondingly, HSV lacking vhs is significantly more immunogenic than virus with intact vhs [130,131]. This may originate from the ability of vhs to impair the production of IFN, cytokines, and chemokines but may also derive from vhs-mediated inhibition of DC maturation, activity, and migration [105,132]. Future studies are needed to determine the underlying mechanisms of the vhs-impaired immune response and the decreased expression of immunoregulatory cytokines.

Finally, the viral proteins ICP34.5 and Us11 inhibit PKR activity. Both PKR and PKR-like endoplasmic-reticulum (ER)-resident kinase (PERK) activity is inhibited by ICP34.5 [61,62,86]. PKR and PERK are otherwise activated after HSV infection resulting in phosphorylation of eIF2α and subsequent halt of protein synthesis. Furthermore, Us11 inhibits dsRNA- and PKR activator protein (PACT)-mediated activation of PKR [63,64]. Collectively, Us11 and ICP34.5 block the activity of PKR and result in sustained levels of protein synthesis. The interference with PKR may, however, potentially also affect the transcriptional activation mediated by PKR.

Recently, ICP34.5 has been added to the list of HSV-induced inhibitors of antiviral signaling via interaction with the signaling molecule TBK1 [85], a protein important for both IRF3 and NF-κB signaling after RLR and TLR stimulation. Why HSV uses three proteins for inhibition of NF-κB and IRF3 signaling (ICP0, ICP27 and ICP34.5) remains to be determined. Several explanations may be given, including cell dependency of evasion strategy employed by the virus and use of different viral proteins at different time-points of infection.

### 5.3. Inhibition of autophagy and intrinsic protection

Autophagy is the process in which the cell engulfs its own cytoplasmic components, which are then degraded in lysosomes. The process is a normal part of cells development and homeostasis. When regulated properly, autophagy participates in maintaining a balance between production and degradation of cellular products and subsequent recycling of the cellular constituents. Recently, the autophagy process has been linked to antimicrobial immunity, playing a protective role in clearance of intracellular parasites, bacteria and viruses [133–135]. In the context of HSV, autophagic degradation of HSV-1 virions has been shown to proceed via a PKR-dependent mechanism that requires the eIF2a kinase signalling pathway [136]. In return HSV has evolved a mechanism to evade the autophagy process via targeting of the essential autophagy protein Beclin-1 with ICP34.5 [66]. Of note, the autophagy process is primarily important for neuroprotection, but does not restrict infection in permissive cell cultures, indicating that the primary function of ICP34.5 in dividing cells is its inhibition of PKR-mediated translational arrest [137].

Recently, another potential antimicrobial pathway was reported to be targeted by HSV via ICP4 and ICP0. The studies showed that HSV downregulates secretory leukocyte proteases inhibitor (SLPI), a low-molecular mass anti-bacteria and anti-viral protein found in mucosal secretions. The downregulation was dependent on ICP0 and ICP4, since viruses deficient in ICP0 or ICP4 were unable to downregulate SLPI [100]. Whether the downregulation of SLPI plays an important role in HSV pathogenesis remains to be determined.

## 6. Concluding remarks

Although some therapeutic and diagnostic improvements for control of HSV have been developed during the last years, the virus is by no means under control. Therefore research on the pathogenesis of HSV and knowledge of overall virus-cell interactions leading to an efficient immune response are still most needed.

The outcome of HSV infections is dependent on the equilibration between virus propagation and an effective immune response. Appropriate expression of IFNs, cytokines, and chemokines is essential for efficient host defense against infection. IFN and cytokines expression is mediated by interaction between the virus PAMPS with cellular PRRs. For HSV, TLR2, TLR3, TLR9, the MAVS pathways and the RNA polymerase III pathway have been identified as sensing the infection and inducing the production of IFN-α/β and cytokines.

HSV has evolved together with its host and developed mechanisms to overcome the effects of the immune response. The countermeasures are directed at several antiviral host defenses, in particular the cytokine and IFN system but also the complement system and antigen-dependent responses (Table 1, Figure 3). These countermeasures are essential for effective virus propagation and to secure an environment suited for virus replication and establishment of latency. Therefore investigations into the viral immune evasion strategies will hopefully provide additional understanding of the innate and adaptive immune mechanisms and reveal viral components essential for HSV. The new understanding of HSV infection and immunopathology might help in development of improved treatments and in design of vaccines.

## Figures and Tables

**Table 1.** Immune evasion strategies employed by HSV.

**Viral protein**	**Effect**	**Mechanism**	**References**
**Inhibition of IFN and cytokine signalling and IFN function**			
ICP0	Enhanced resistance to IFN	Modification of IRF3 and IRF7 activation	[70–74]
ICP27	Decreased IFN and cytokine expression	Reduces IRF3 and NF-κB activation	[27]
Us3	Decreased ISG expression (Mx) and reduced IRF3 activation	*n.d.*	[79]
vhs	Inhibition of IFN-α/β production	Potentially because of reduced IRF7 activation	[80–82]
vhs	Inhibition of JAK/STAT signalling	Induction of SOCS3, repression of STAT1 activation	[82,83]
ICP27	Inhibition of IFN signalling	Decreased STAT1 activation and translocation to the nucleus	[78]
vhs	Suppression of proinflammatory cytokines, IFNs, and chemokines	*n.d.*	[84]
ICP34.5	Suppression of antiviral genes	Inhibition of IRF3 activation via interaction with TBK1	[85]
ICP34.5	Inhibition of PKR and PERK activity	Reverses the PKR and PERK-induced phosphorylation of eIF2α	[61,62,86]
Us11	Inhibition of dsRNA-dependent and PACT-mediated activation of PKR	Binds to dsRNA	[63]
		Binds to PKR	[64]
Us11	Inhibition of 2′–5′ OAS	Binding to dsRNA (dsRNA binding domain of Us11 essential)	[67]
2′–5′A analog	Inhibition of the 2′–5′ OAS/ RNAse L system	2′–5′ A analogue	[69]
ICP0	Inhibition of RNAseL-independent rRNA degradation	*n.d.*	[68]

**Inhibition of host gene expression**			
ICP0	Inhibition of TLR-induced JNK and NF-κB activation	Recruitment of USP7 binding to TRAF6 and IKKγ	[87]
ICP27	Inhibition of splicing	Interacts with spliceosome components	[88–90]
ICP27	Reduction of mRNA stability	*n.d.*	[91]
VP16 and vhs	RNA degradation	*n.d.*	[92]
ICP0	Cell cycle arrest and disturbed cellular gene expression	Upregulation of p53-responsive genes.	[93]
*Unknown*	Inhibition of NFAT activation	*n.d.*	[94]

**Inhibition of apoptosis**			
ICP4	Inhibition of apoptosis	*n.d.*	[95]
gJ	Inhibition of apoptosis	Inhibition of caspase activation	[96]
ICP27	Inhibition of apoptosis	*n.d.*	[97]
ICP34.5	Inhibition of apoptosis	Inhibition of PKR activity	[62]
	Inhibition of CTL-induced cell death (apoptosis)	Downregulation of cell surface Fas ligand	[98,99]

**Inhibition of autophagy and anti-microbial proteins**			
ICP0 and ICP0	Inhibition of SLPI	*n.d.*	[100]
ICP34.5	Inhibition of autophagy	Targeting of Beclin-1	[66]

**Inhibition of complement, antigen presentation and APC function**			
gC	Inhibition of complement	Binds to complement factor C3	[101,102]
gE/gI complex	Blocking of Fc-mediated activities, including complement activation and ADCC	Binds to Fc domain of IgG	[103]
vhs	Inhibition of DC maturation and reduced cytokine production	*n.d.*	[104,105]
ICP47	Inhibition of antigen presentation by MHC I	Interferes with TAP1/TAP2	[106–108]
vhs	Inhibition of antigen presentation by MHC I and MHC II	Interferes with MHC I transport. Reduces levels of MHC II	[109–111]
gB	Inhibition of MHC II-mediated antigen presentation	Inhibited expression of invariant chain and interacts with HLA-DR and HLA-DM	[112]

**Abbreviations:** ADCC, antibody-dependent cellular cytotoxicity; CTL, cytotoxic T lymphocyte; DC, dendritic cell; HLA, human leukocyte antigen; ICP, infected cell protein; JAK, janus kinase; MHC, major histocompability complex; n.d., not determined; NFAT, nuclear factor of activated T cell; PACT, PKR-activating protein; PERK, PKR-like endoplasmic-reticulum (ER)-resident kinase; PKR, dsRNA-activated protein kinase R; SLPI, secretory leukocyte protease inhibitor; SOCS, suppressor of cytokine signalling; STAT, signal transducer and activator of transcription; TAP, transporter accociated with antigen presentation; vhs, virion-host shutoff protein.

## References

[1] Roizman B, Knipe DM, Whitley RJ, Knipe DM, Howley PM, Griffin DE, Lamb RA, Martin MA, Roizman B, Straus SE (2007). Herpes simplex virus. Fields Virology.

[2] Pinto AJ, Morahan PS, Brinton M, Stewart D, Gavin E (1990). Comparative therapeutic efficacy of recombinant interferons-alpha, -beta, and -gamma against alphatogavirus, bunyavirus, flavivirus, and herpesvirus infections. J Interferon Res.

[3] Leib DA, Harrison TE, Laslo KM, Machalek MA, Moorman NJ, Virgin HW (1999). Interferons regulate the phenotype of wild-type and mutant herpes simplex viruses *in vivo*. J Exp Med.

[4] Dupuis S, Jouanguy E, Al Hajjar S, Fieschi C, Al Mohsen IZ, Al Jumaah S, Yang K, Chapgier A, Eidenschenk C, Eid P, Al Ghonaium A, Tufenkeji H, Frayha H, Al Gazlan S, Al Rayes H, Schreiber RD, Gresser I, Casanova JL (2003). Impaired response to interferon-alpha/beta and lethal viral disease in human STAT1 deficiency. Nat Genet.

[5] Ank N, Iversen MB, Bartholdy C, Staeheli P, Hartmann R, Jensen UB, Dagnaes-Hansen F, Thomsen AR, Chen Z, Haugen H, Klucher K, Paludan SR (2008). An important role for type III Interferon (IFN-lambda/IL-28) in TLR-induced antiviral activity. J Immunol.

[6] Takeuchi O, Akira S (2009). Innate immunity to virus infection. Immunol Rev.

[7] Takaoka A, Wang Z, Choi MK, Yanai H, Negishi H, Ban T, Lu Y, Miyagishi M, Kodama T, Honda K, Ohba Y, Taniguchi T (2007). DAI (DLM-1/ZBP1) is a cytosolic DNA sensor and an activator of innate immune response. Nature.

[8] Ablasser A, Bauernfeind F, Hartmann G, Latz E, Fitzgerald KA, Hornung V (2009). RIG-I-dependent sensing of poly(dA:dT) through the induction of an RNA polymerase III-transcribed RNA intermediate. Nat Immunol.

[9] Chiu YH, Macmillan JB, Chen ZJ (2009). RNA Polymerase III Detects Cytosolic DNA and Induces Type I Interferons through the RIG-I Pathway. Cell.

[10] Kurt-Jones EA, Chan M, Zhou S, Wang J, Reed G, Bronson R, Arnold MM, Knipe DM, Finberg RW (2004). Herpes simplex virus 1 interaction with Toll-like receptor 2 contributes to lethal encephalitis. Proc Natl Acad Sci USA.

[11] Aravalli RN, Hu S, Rowen TN, Palmquist JM, Lokensgard JR (2005). Cutting Edge: TLR2-Mediated Proinflammatory Cytokine and Chemokine Production by Microglial Cells in Response to Herpes Simplex Virus. J Immunol.

[12] Reske A, Pollara G, Krummenacher C, Katz DR, Chain BM (2008). Glycoprotein-dependent and TLR2-independent innate immune recognition of herpes simplex virus-1 by dendritic cells. J Immunol.

[13] Lund J, Sato A, Akira S, Medzhitov R, Iwasaki A (2003). Toll-like receptor 9-mediated recognition of Herpes simplex virus-2 by plasmacytoid dendritic cells. J Exp Med.

[14] Hochrein H, Schlatter B, O'Keeffe M, Wagner C, Schmitz F, Schiemann M, Bauer S, Suter M, Wagner H (2004). Herpes simplex virus type-1 induces IFN-alpha production via Toll-like receptor 9-dependent and -independent pathways. Proc Natl Acad Sci USA.

[15] Malmgaard L, Melchjorsen J, Bowie AG, Mogensen SC, Paludan SR (2004). Viral activation of macrophages through TLR-dependent and -independent pathways. J Immunol.

[16] Rasmussen SB, Sorensen LN, Malmgaard L, Ank N, Baines JD, Chen ZJ, Paludan SR (2007). Type I IFN production during herpes simplex virus infection is controlled by cell-type specific viral recognition through TLR9, the MAVS pathway, and novel recognition systems. J Virol.

[17] Zhang SY, Jouanguy E, Ugolini S, Smahi A, Elain G, Romero P, Segal D, Sancho-Shimizu V, Lorenzo L, Puel A, Picard C, Chapgier A, Plancoulaine S, Titeux M, Cognet C, von Bernuth H, Ku CL, Casrouge A, Zhang XX, Barreiro L, Leonard J, Hamilton C, Lebon P, Heron B, Vallee L, Quintana-Murci L, Hovnanian A, Rozenberg F, Vivier E, Geissmann F, Tardieu M, Abel L, Casanova JL (2007). TLR3 deficiency in patients with herpes simplex encephalitis. Science.

[18] Dolan A, Jamieson FE, Cunningham C, Barnett BC, McGeoch DJ (1998). The genome sequence of herpes simplex virus type 2. J Virol.

[19] Fruh K, Gruhler A, Krishna RM, Schoenhals GJ (1999). A comparison of viral immune escape strategies targeting the MHC class I assembly pathway. Immunol Rev.

[20] Homa FL, Brown JC (1997). Capsid assembly and DNA packaging in herpes simplex virus. Rev Med Virol.

[21] Ellermann-Eriksen S (1993). Autocrine secretion of interferon-alpha/beta and tumour necrosis factor-alpha synergistically activates mouse macrophages after infection with herpes simplex virus type 2. J Gen Virol.

[22] Mittnacht S, Straub P, Kirchner H, Jacobsen H (1988). Interferon treatment inhibits onset of herpes simplex virus immediate-early transcription. Virology.

[23] Oberman F, Panet A (1988). Inhibition of transcription of herpes simplex virus immediate early genes in interferon-treated human cells. J Gen Virol.

[24] Sainz B, Halford WP (2002). Alpha/Beta interferon and gamma interferon synergize to inhibit the replication of herpes simplex virus type 1. J Virol.

[25] Vollstedt S, Arnold S, Schwerdel C, Franchini M, Alber G, Di Santo JP, Ackermann M, Suter M (2004). Interplay between alpha/beta and gamma interferons with B, T, and natural killer cells in the defense against herpes simplex virus type 1. J Virol.

[26] Gresser I, Tovey MG, Maury C, Bandu MT (1976). Role of interferon in the pathogenesis of virus diseases in mice as demonstrated by the use of anti-interferon serum. II. Studies with herpes simplex, Moloney sarcoma, vesicular stomatitis, Newcastle disease, and influenza viruses. J Exp Med.

[27] Melchjorsen J, Siren J, Julkunen I, Paludan SR, Matikainen S (2006). Induction of cytokine expression by herpes simplex virus in human monocyte-derived macrophages and dendritic cells is dependent on virus replication and is counteracted by ICP27 targeting NF-kappaB and IRF-3. J Gen Virol.

[28] Der SD, Zhou A, Williams BR, Silverman RH (1998). Identification of genes differentially regulated by interferon alpha, beta, or gamma using oligonucleotide arrays. Proc Natl Acad Sci USA.

[29] Khabar KS, Dhalla M, Siddiqui Y, Zhou A, Al Ahdal MN, Der SD, Silverman RH, Williams BR (2000). Effect of deficiency of the double-stranded RNA-dependent protein kinase, PKR, on antiviral resistance in the presence or absence of ribonuclease L: HSV-1 replication is particularly sensitive to deficiency of the major IFN-mediated enzymes. J Interferon Cytokine Res.

[30] Melchjorsen J, Pedersen FS, Mogensen SC, Paludan SR (2002). Herpes simplex virus selectively induces expression of the CC Chemokine RANTES/CCL5 in macrophages through a mechanism dependent on PKR and ICP0. J Virol.

[31] Zheng X, Silverman RH, Zhou A, Goto T, Kwon BS, Kaufman HE, Hill JM (2001). Increased severity of HSV-1 keratitis and mortality in mice lacking the 2-5A-dependent RNase L gene. Invest Ophthalmol Vis Sci.

[32] Carr DJ, Al khatib K, James CM, Silverman R (2003). Interferon-beta suppresses herpes simplex virus type 1 replication in trigeminal ganglion cells through an RNase L-dependent pathway. J Neuroimmunol.

[33] Fujihara M, Milligan JR, Kaji A (1989). Effect of 2′,5′-oligoadenylate on herpes simplex virus-infected cells and preventive action of 2′,5′-oligoadenylate on the lethal effect of HSV-2. J Interferon Res.

[34] Neville LF, Mathiak G, Bagasra O (1997). The immunobiology of interferon-gamma inducible protein 10 kD (IP-10): a novel, pleiotropic member of the C-X-C chemokine superfamily. Cytokine Growth Factor Rev.

[35] Levy DE, Marie I, Smith E, Prakash A (2002). Enhancement and diversification of IFN induction by IRF-7-mediated positive feedback. J Interferon Cytokine Res.

[36] Miettinen M, Sareneva T, Julkunen I, Matikainen S (2001). IFNs activate toll-like receptor gene expression in viral infections. Genes Immun.

[37] Siren J, Pirhonen J, Julkunen I, Matikainen S (2005). IFN-alpha regulates TLR-dependent gene expression of IFN-alpha, IFN-beta, IL-28, and IL-29. J Immunol.

[38] Foy E, Li K, Sumpter R, Loo YM, Johnson CL, Wang C, Fish PM, Yoneyama M, Fujita T, Lemon SM, Gale M (2005). Control of antiviral defenses through hepatitis C virus disruption of retinoic acid-inducible gene-I signaling. Proc Natl Acad Sci USA.

[39] Kang DC, Gopalkrishnan RV, Wu Q, Jankowsky E, Pyle AM, Fisher PB (2002). mda-5: An interferon-inducible putative RNA helicase with double-stranded RNA-dependent ATPase activity and melanoma growth-suppressive properties. Proc Natl Acad Sci USA.

[40] Ank N, West H, Bartholdy C, Eriksson K, Thomsen AR, Paludan SR (2006). Lambda Interferon (IFN-{lambda}), a Type III IFN, Is Induced by Viruses and IFNs and Displays Potent Antiviral Activity against Select Virus Infections. In vivo J Virol.

[41] Kotenko SV, Gallagher G, Baurin VV, Lewis-Antes A, Shen M, Shah NK, Langer JA, Sheikh F, Dickensheets H, Donnelly RP (2003). IFN-lambdas mediate antiviral protection through a distinct class II cytokine receptor complex. Nat Immunol.

[42] Sheppard P, Kindsvogel W, Xu W, Henderson K, Schlutsmeyer S, Whitmore TE, Kuestner R, Garrigues U, Birks C, Roraback J, Ostrander C, Dong D, Shin J, Presnell S, Fox B, Haldeman B, Cooper E, Taft D, Gilbert T, Grant FJ, Tackett M, Krivan W, McKnight G, Clegg C, Foster D, Klucher KM (2003). IL-28, IL-29 and their class II cytokine receptor IL-28R. Nat Immunol.

[43] Coccia EM, Severa M, Giacomini E, Monneron D, Remoli ME, Julkunen I, Cella M, Lande R, Uze G (2004). Viral infection and Toll-like receptor agonists induce a differential expression of type I and lambda interferons in human plasmacytoid and monocyte-derived dendritic cells. Eur J Immunol.

[44] Karupiah G, Xie QW, Buller RM, Nathan C, Duarte C, MacMicking JD (1993). Inhibition of viral replication by interferon-gamma-induced nitric oxide synthase. Science.

[45] Chesler DA, Reiss CS (2002). The role of IFN-gamma in immune responses to viral infections of the central nervous system. Cytokine Growth Factor Rev.

[46] Vollstedt S, Franchini M, Alber G, Ackermann M, Suter M (2001). Interleukin-12- and gamma interferon-dependent innate immunity are essential and sufficient for long-term survival of passively immunized mice infected with herpes simplex virus type 1. J Virol.

[47] Sen GC (2001). Viruses and interferons. AnnuRevMicrobiol.

[48] Malmgaard L, Paludan SR (2003). Interferon (IFN)-alpha/beta, interleukin (IL)-12 and IL-18 coordinately induce production of IFN-gamma during infection with herpes simplex virus type 2. J Gen Virol.

[49] Munder M, Mallo M, Eichmann K, Modolell M (1998). Murine macrophages secrete interferon gamma upon combined stimulation with interleukin (IL)-12 and IL-18: A novel pathway of autocrine macrophage activation. J Exp Med.

[50] Schindler H, Lutz MB, Rollinghoff M, Bogdan C (2001). The production of IFN-gamma by IL-12/IL-18-activated macrophages requires STAT4 signaling and is inhibited by IL-4. J Immunol.

[51] Stober D, Schirmbeck R, Reimann J (2001). IL-12/IL-18-dependent IFN-gamma release by murine dendritic cells. J Immunol.

[52] Kodukula P, Liu T, Rooijen NV, Jager MJ, Hendricks RL (1999). Macrophage control of herpes simplex virus type 1 replication in the peripheral nervous system. J Immunol.

[53] Paludan SR, Mogensen SC (2001). Virus-cell interactions regulating induction of tumor necrosis factor alpha production in macrophages infected with herpes simplex virus. J Virol.

[54] Malmgaard L, Paludan SR, Mogensen SC, Ellermann-Eriksen S (2000). Herpes simplex virus type 2 induces secretion of IL-12 by macrophages through a mechanism involving NF-kappaB. J Gen Virol.

[55] Imaizumi T, Hatakeyama M, Yamashita K, Yoshida H, Ishikawa A, Taima K, Satoh K, Mori F, Wakabayashi K (2004). Interferon-gamma induces retinoic acid-inducible gene-I in endothelial cells. Endothelium.

[56] Seth RB, Sun L, Chen ZJ (2006). Antiviral innate immunity pathways. Cell Res.

[57] Rasmussen SB, Jensen SB, Nielsen C, Quartin E, Kato H, Chen ZJ, Silverman RH, Akira S, Paludan SR (2009). Herpes simplex virus infection is sensed by both Toll-like receptors and retinoic acid-inducible gene- like receptors, which synergize to induce type I interferon production. J Gen Virol.

[58] Patel A, Hanson J, McLean TI, Olgiate J, Hilton M, Miller WE, Bachenheimer SL (1998). Herpes simplex type 1 induction of persistent NF-kappa B nuclear translocation increases the efficiency of virus replication. Virology.

[59] Leonard WJ, O'Shea JJ (1998). Jaks and STATs: biological implications. Annu Rev Immunol.

[60] Pasieka TJ, Lu B, Leib DA (2008). Enhanced pathogenesis of an attenuated herpes simplex virus for mice lacking Stat1. J Virol.

[61] Chou J, Chen JJ, Gross M, Roizman B (1995). Association of a M(r) 90,000 phosphoprotein with protein kinase PKR in cells exhibiting enhanced phosphorylation of translation initiation factor eIF-2 alpha and premature shutoff of protein synthesis after infection with gamma 134.5- mutants of herpes simplex virus 1. Proc Natl Acad Sci USA.

[62] He B, Gross M, Roizman B (1997). The gamma(1)34.5 protein of herpes simplex virus 1 complexes with protein phosphatase 1alpha to dephosphorylate the alpha subunit of the eukaryotic translation initiation factor 2 and preclude the shutoff of protein synthesis by double-stranded RNA-activated protein kinase. Proc Natl Acad Sci USA.

[63] Poppers J, Mulvey M, Khoo D, Mohr I (2000). Inhibition of PKR activation by the proline-rich RNA binding domain of the herpes simplex virus type 1 Us11 protein. J Virol.

[64] Peters GA, Khoo D, Mohr I, Sen GC (2002). Inhibition of PACT-mediated activation of PKR by the herpes simplex virus type 1 Us11 protein. J Virol.

[65] Mulvey M, Camarena V, Mohr I (2004). Full resistance of herpes simplex virus type 1-infected primary human cells to alpha interferon requires both the Us11 and gamma(1)34.5 gene products. J Virol.

[66] Orvedahl A, Alexander D, Talloczy Z, Sun Q, Wei Y, Zhang W, Burns D, Leib DA, Levine B (2007). HSV-1 ICP34.5 confers neurovirulence by targeting the Beclin 1 autophagy protein. Cell Host Microbe.

[67] Sanchez R, Mohr I (2007). Inhibition of cellular 2′–5′ oligoadenylate synthetase by the herpes simplex virus type 1 Us11 protein. J Virol.

[68] Sobol PT, Mossman KL (2006). ICP0 Prevents RNase L-Independent rRNA Cleavage in Herpes Simplex Virus Type 1-Infected Cells. J Virol.

[69] Cayley PJ, Davies JA, McCullagh KG, Kerr IM (1984). Activation of the ppp(A2′p)nA system in interferon-treated, herpes simplex virus-infected cells and evidence for novel inhibitors of the ppp(A2′p)nA-dependent RNase. EurJBiochem.

[70] Mossman KL, Saffran HA, Smiley JR (2000). Herpes simplex virus ICP0 mutants are hypersensitive to interferon. J Virol.

[71] Harle P, Sainz B, Carr DJ, Halford WP (2002). The immediate-early protein, ICP0, is essential for the resistance of herpes simplex virus to interferon-alpha/beta. Virology.

[72] Lin R, Noyce RS, Collins SE, Everett RD, Mossman KL (2004). The herpes simplex virus ICP0 RING finger domain inhibits IRF3 and IRF7-mediated activation of interferon-stimulated genes. J Virol.

[73] Melroe GT, DeLuca NA, Knipe DM (2004). Herpes simplex virus 1 has multiple mechanisms for blocking virus-induced interferon production. J Virol.

[74] Eidson KM, Hobbs WE, Manning BJ, Carlson P, DeLuca NA (2002). Expression of herpes simplex virus ICP0 inhibits the induction of interferon-stimulated genes by viral Infection. J Virol.

[75] Halford WP, Weisend C, Grace J, Soboleski M, Carr DJ, Balliet JW, Imai Y, Margolis TP, Gebhardt BM (2006). ICP0 antagonizes Stat 1-dependent repression of herpes simplex virus: implications for the regulation of viral latency. Virol J.

[76] Everett RD, Orr A (2009). Herpes simplex virus type 1 regulatory protein ICP0 aids infection in cells with a preinduced interferon response but does not impede interferon-induced gene induction. J Virol.

[77] Everett RD, Young DF, Randall RE, Orr A (2008). STAT-1- and IRF-3-dependent pathways are not essential for repression of ICP0-null mutant herpes simplex virus type 1 in human fibroblasts. J Virol.

[78] Johnson KE, Song B, Knipe DM (2008). Role for herpes simplex virus 1 ICP27 in the inhibition of type I interferon signaling. Virology.

[79] Peri P, Mattila RK, Kantola H, Broberg E, Karttunen HS, Waris M, Vuorinen T, Hukkanen V (2008). Herpes simplex virus type 1 Us3 gene deletion influences toll-like receptor responses in cultured monocytic cells. Virol J.

[80] Murphy JA, Duerst RJ, Smith TJ, Morrison LA (2003). Herpes simplex virus type 2 virion host shutoff protein regulates alpha/beta interferon but not adaptive immune responses during primary infection *in vivo*. J Virol.

[81] Duerst RJ, Morrison LA (2004). Herpes simplex virus 2 virion host shutoff protein interferes with type I interferon production and responsiveness. Virology.

[82] Yokota S, Yokosawa N, Okabayashi T, Suzutani T, Miura S, Jimbow K, Fujii N (2004). Induction of suppressor of cytokine signaling-3 by herpes simplex virus type 1 contributes to inhibition of the interferon signaling pathway. J Virol.

[83] Yokota S, Yokosawa N, Kubota T, Suzutani T, Yoshida I, Miura S, Jimbow K, Fujii N (2001). Herpes simplex virus type 1 suppresses the interferon signaling pathway by inhibiting phosphorylation of STATs and janus kinases during an early infection stage. Virology.

[84] Suzutani T, Nagamine M, Shibaki T, Ogasawara M, Yoshida I, Daikoku T, Nishiyama Y, Azuma M (2000). The role of the UL41 gene of herpes simplex virus type 1 in evasion of non-specific host defence mechanisms during primary infection. J Gen Virol.

[85] Verpooten D, Ma Y, Hou S, Yan Z, He B (2009). Control of TANK-binding kinase 1-mediated signaling by the gamma(1)34.5 protein of herpes simplex virus 1. J Biol Chem.

[86] Cheng G, Feng Z, He B (2005). Herpes simplex virus 1 infection activates the endoplasmic reticulum resident kinase PERK and mediates eIF-2alpha dephosphorylation by the gamma(1)34.5 protein. J Virol.

[87] Daubeuf S, Singh D, Tan Y, Liu H, Federoff HJ, Bowers WJ, Tolba K (2009). HSV ICP0 recruits USP7 to modulate TLR-mediated innate response. Blood.

[88] Hardy WR, Sandri-Goldin RM (1994). Herpes simplex virus inhibits host cell splicing, and regulatory protein ICP27 is required for this effect. J Virol.

[89] Sciabica KS, Dai QJ, Sandri-Goldin RM (2003). ICP27 interacts with SRPK1 to mediate HSV splicing inhibition by altering SR protein phosphorylation. EMBO J.

[90] Bryant HE, Wadd SE, Lamond AI, Silverstein SJ, Clements JB (2001). Herpes simplex virus IE63 (ICP27) protein interacts with spliceosome-associated protein 145 and inhibits splicing prior to the first catalytic step. J Virol.

[91] Mogensen TH, Melchjorsen J, Malmgaard L, Casola A, Paludan SR (2004). Suppression of proinflammatory cytokine expression by herpes simplex virus type 1. J Virol.

[92] Strand SS, Leib DA (2004). Role of the VP16-binding domain of vhs in viral growth, host shutoff activity, and pathogenesis. J Virol.

[93] Hobbs WE, DeLuca NA (1999). Perturbation of cell cycle progression and cellular gene expression as a function of herpes simplex virus ICP0. J Virol.

[94] Scott ES, Malcomber S, O'Hare P (2001). Nuclear translocation and activation of the transcription factor NFAT is blocked by herpes simplex virus infection. J Virol.

[95] Leopardi R, Roizman B (1996). The herpes simplex virus major regulatory protein ICP4 blocks apoptosis induced by the virus or by hyperthermia. Proc Natl Acad Sci USA.

[96] Jerome KR, Chen Z, Lang R, Torres MR, Hofmeister J, Smith S, Fox R, Froelich CJ, Corey L (2001). HSV and glycoprotein J inhibit caspase activation and apoptosis induced by granzyme B or Fas. J Immunol.

[97] Aubert M, Blaho JA (1999). The herpes simplex virus type 1 regulatory protein ICP27 is required for the prevention of apoptosis in infected human cells. J Virol.

[98] Sieg S, Yildirim Z, Smith D, Kayagaki N, Yagita H, Huang Y, Kaplan D (1996). Herpes simplex virus type 2 inhibition of Fas ligand expression. J Virol.

[99] Sieg S, Huang Y, Kaplan D (1997). Viral regulation of CD95 expression and apoptosis in T lymphocytes. J Immunol.

[100] Fakioglu E, Wilson SS, Mesquita PM, Hazrati E, Cheshenko N, Blaho JA, Herold BC (2008). Herpes simplex virus downregulates secretory leukocyte protease inhibitor: a novel immune evasion mechanism. J Virol.

[101] Friedman HM, Cohen GH, Eisenberg RJ, Seidel CA, Cines DB (1984). Glycoprotein C of herpes simplex virus 1 acts as a receptor for the C3b complement component on infected cells. Nature.

[102] Lubinski J, Wang L, Mastellos D, Sahu A, Lambris JD, Friedman HM (1999). *In vivo* role of complement-interacting domains of herpes simplex virus type 1 glycoprotein gC. J Exp Med.

[103] Lubinski J, Nagashunmugam T, Friedman HM (1998). Viral interference with antibody and complement. Semin Cell Dev Biol.

[104] Salio M, Cella M, Suter M, Lanzavecchia A (1999). Inhibition of dendritic cell maturation by herpes simplex virus. Eur J Immunol.

[105] Samady L, Costigliola E, MacCormac L, McGrath Y, Cleverley S, Lilley CE, Smith J, Latchman DS, Chain B, Coffin RS (2003). Deletion of the virion host shutoff protein (vhs) from herpes simplex virus (HSV) relieves the viral block to dendritic cell activation: potential of vhs- HSV vectors for dendritic cell-mediated immunotherapy. J Virol.

[106] Fruh K, Ahn K, Djaballah H, Sempe P, van Endert PM, Tampe R, Peterson PA, Yang Y (1995). A viral inhibitor of peptide transporters for antigen presentation. Nature.

[107] Hill A, Jugovic P, York I, Russ G, Bennink J, Yewdell J, Ploegh H, Johnson D (1995). Herpes simplex virus turns off the TAP to evade host immunity. Nature.

[108] Ahn K, Meyer TH, Uebel S, Sempe P, Djaballah H, Yang Y, Peterson PA, Fruh K, Tampe R (1996). Molecular mechanism and species specificity of TAP inhibition by herpes simplex virus ICP47. EMBO J.

[109] Hill AB, Barnett BC, McMichael AJ, McGeoch DJ (1994). HLA class I molecules are not transported to the cell surface in cells infected with herpes simplex virus types 1 and 2. J Immunol.

[110] Tigges MA, Leng S, Johnson DC, Burke RL (1996). Human herpes simplex virus (HSV)-specific CD8+ CTL clones recognize HSV-2-infected fibroblasts after treatment with IFN-gamma or when virion host shutoff functions are disabled. J Immunol.

[111] Trgovcich J, Johnson D, Roizman B (2002). Cell surface major histocompatibility complex class II proteins are regulated by the products of the gamma(1)34.5 and U(L)41 genes of herpes simplex virus 1. J Virol.

[112] Neumann J, Eis-Hubinger AM, Koch N (2003). Herpes simplex virus type 1 targets the MHC class II processing pathway for immune evasion. J Immunol.

[113] Stingley SW, Ramirez JJ, Aguilar SA, Simmen K, Sandri-Goldin RM, Ghazal P, Wagner EK (2000). Global analysis of herpes simplex virus type 1 transcription using an oligonucleotide-based DNA microarray. J Virol.

[114] Kim JC, Lee SY, Kim SY, Kim JK, Kim HJ, Lee HM, Choi MS, Min JS, Kim MJ, Choi HS, Ahn JK (2008). HSV-1 ICP27 suppresses NF-kappaB activity by stabilizing IkappaBalpha. FEBS Lett.

[115] Hargett D, McLean T, Bachenheimer SL (2005). Herpes Simplex Virus ICP27 Activation of Stress Kinases JNK and p38. J Virol.

[116] Gillis PA, Okagaki LH, Rice SA (2009). Herpes simplex virus type 1 ICP27 induces p38 mitogen-activated protein kinase signaling and apoptosis in HeLa cells. J Virol.

[117] Hargett D, Rice S, Bachenheimer SL (2006). Herpes simplex virus type 1 ICP27-dependent activation of NF-kappaB. J Virol.

[118] Smiley JR (2004). Herpes simplex virus virion host shutoff protein: immune evasion mediated by a viral RNase. J Virol.

[119] Honda K, Yanai H, Negishi H, Asagiri M, Sato M, Mizutani T, Shimada N, Ohba Y, Takaoka A, Yoshida N, Taniguchi T (2005). IRF-7 is the master regulator of type-I interferon-dependent immune responses. Nature.

[120] Frey KG, Ahmed CM, Dabelic R, Jager LD, Noon-Song EN, Haider SM, Johnson HM, Bigley NJ (2009). HSV-1-Induced SOCS-1 Expression in Keratinocytes: Use of a SOCS-1 Antagonist to Block a Novel Mechanism of Viral Immune Evasion. J Immunol.

[121] Mossman KL, Macgregor PF, Rozmus JJ, Goryachev AB, Edwards AM, Smiley JR (2001). Herpes simplex virus triggers and then disarms a host antiviral response. J Virol.

[122] Spencer CA, Dahmus ME, Rice SA (1997). Repression of host RNA polymerase II transcription by herpes simplex virus type 1. J Virol.

[123] Collins SE, Noyce RS, Mossman KL (2004). Innate cellular response to virus particle entry requires IRF3 but not virus replication. J Virol.

[124] Hardwicke MA, Sandri-Goldin RM (1994). The herpes simplex virus regulatory protein ICP27 contributes to the decrease in cellular mRNA levels during infection. J Virol.

[125] McCarthy AM, McMahan L, Schaffer PA (1989). Herpes simplex virus type 1 ICP27 deletion mutants exhibit altered patterns of transcription and are DNA deficient. J Virol.

[126] Song B, Yeh KC, Liu J, Knipe DM (2001). Herpes simplex virus gene products required for viral inhibition of expression of G1-phase functions. Virology.

[127] Zhou C, Knipe DM (2002). Association of herpes simplex virus type 1 ICP8 and ICP27 proteins with cellular RNA polymerase II holoenzyme. J Virol.

[128] Kawaguchi Y, Bruni R, Roizman B (1997). Interaction of herpes simplex virus 1 alpha regulatory protein ICP0 with elongation factor 1delta: ICP0 affects translational machinery. J Virol.

[129] Sin JI, Kim JJ, Pachuk C, Satishchandran C, Weiner DB (2000). DNA vaccines encoding interleukin-8 and RANTES enhance antigen-specific Th1-type CD4(+) T-cell-mediated protective immunity against herpes simplex virus type 2. In vivo J Virol.

[130] Walker J, Leib DA (1998). Protection from primary infection and establishment of latency by vaccination with a herpes simplex virus type 1 recombinant deficient in the virion host shutoff (vhs) function. Vaccine.

[131] Geiss BJ, Smith TJ, Leib DA, Morrison LA (2000). Disruption of virion host shutoff activity improves the immunogenicity and protective capacity of a replication-incompetent herpes simplex virus type 1 vaccine strain. J Virol.

[132] Prechtel AT, Turza NM, Kobelt DJ, Eisemann JI, Coffin RS, McGrath Y, Hacker C, Ju X, Zenke M, Steinkasserer A (2005). Infection of mature dendritic cells with herpes simplex virus type 1 dramatically reduces lymphoid chemokine-mediated migration. J Gen Virol.

[133] Lee HK, Iwasaki A (2008). Autophagy and antiviral immunity. CurrOpinImmunol.

[134] Espert L, Codogno P, Biard-Piechaczyk M (2007). Involvement of autophagy in viral infections: antiviral function and subversion by viruses. J Mol Med.

[135] Levine B, Deretic V (2007). Unveiling the roles of autophagy in innate and adaptive immunity. Nat Rev Immunol.

[136] Talloczy Z, Virgin HW, Levine B (2006). PKR-dependent autophagic degradation of herpes simplex virus type 1. Autophagy.

[137] Alexander DE, Leib DA (2008). Xenophagy in herpes simplex virus replication and pathogenesis. Autophagy.

